# Antithrombotic Therapy in Acute Coronary Syndrome Patients with End-Stage Renal Disease: Navigating Efficacy and Safety

**DOI:** 10.3390/jcm14113956

**Published:** 2025-06-03

**Authors:** Tarek Abdeldayem, Visvesh Jeyalan, Afzal Hayat, Saif Memon, Mohamed Farag, Mohaned Egred

**Affiliations:** Cardiothoracic Centre, Freeman Hospital, Newcastle-upon-Tyne NE4 5PL, UK; v.jeyalan@nhs.net (V.J.); afzal.hayat@nhs.net (A.H.); saif.memon@nhs.net (S.M.); mohamedfarag@nhs.net (M.F.); m.egred@nhs.net (M.E.)

**Keywords:** anti-thrombotic therapy, end-stage renal disease, dialysis, acute coronary syndrome

## Abstract

Cardiovascular disease is the primary cause of mortality and morbidity in patients with chronic kidney disease (CKD), particularly those with end-stage renal disease (ESRD) undergoing hemodialysis. This paper examines the challenges of managing acute coronary syndrome (ACS) in ESRD patients, focusing on the delicate balance between thrombotic and bleeding risks. The review explores the mechanisms underlying the increased thrombotic risk in ESRD, including elevated platelet aggregation, endothelial dysfunction, and alterations in coagulation factors. Paradoxically, ESRD patients also exhibit higher bleeding tendencies due to platelet dysfunction and other uremia-related factors. The efficacy and safety of various antiplatelet therapies, including aspirin and P2Y12 inhibitors, are evaluated in this population. While potent P2Y12 inhibitors such as ticagrelor and prasugrel have demonstrated potential in reducing ischemic events, they are associated with an increased bleeding risk. The optimal duration of anti-platelet therapy (DAPT) in ESRD patients remains controversial, with studies suggesting potential benefits of prolonged DAPT but also increased bleeding risk. This review underscores the necessity for further research and patient inclusion in clinical trials to establish evidence-based guidelines for tailoring antithrombotic therapy in this high-risk population.

## 1. Introduction

Cardiovascular disease is the primary cause of mortality and morbidity for individuals with chronic kidney disease (CKD). In addition to common risk factors for coronary artery disease (CAD), such as diabetes, hypercholesterolemia, and hypertension, CKD patients are also exposed to cardiovascular risks associated with uremia, including inflammation, oxidative stress, and abnormal calcium-phosphorus metabolism [1]. The risk of cardiovascular complications increases as renal function declines. For instance, individuals with CKD and an estimated glomerular filtration rate (eGFR) below 45 mL/min/M2 are three times more likely to experience acute myocardial infarction as their first indication of CAD compared to those with normal kidney function [2]. Furthermore, CKD patients in stages G3a to G4 (eGFR of 15–60 mL/min/1.73 m^2^) face approximately two and three times the risk of cardiovascular mortality, respectively, in comparison to those without CKD. It is worth noting that CKD patients are at a higher risk of developing cardiovascular disease than progressing to end-stage renal disease (ESRD) [3].

Chronic kidney disease is defined by kidney damage and function levels, regardless of the underlying cause. It is classified into five stages, from Stage 1, with a normal estimated glomerular filtration rate, to Stage 5, with an estimated glomerular filtration rate <15 mL/min/1.73 m^2^ or hemodialysis [4,5]. Hemodialysis patients with end-stage renal disease are susceptible to coronary artery disease, with prevalence from 30% to 60% [5]. A study showed even asymptomatic individuals with ESRD had 41% prevalence of obstructive coronary artery disease. Hemodialysis patients face a ≥4-fold higher risk of thrombotic cardiovascular events, like acute myocardial infarction, compared to the general population [1,4].

Despite significant advancements in percutaneous coronary intervention (PCI), which serves as the primary treatment modality for acute coronary syndrome (ACS) [6], patients with end-stage renal disease (ESRD) face unique challenges. These challenges are primarily due to a higher prevalence of comorbidities, particularly diabetes mellitus. Consequently, these patients experience an increased incidence of ischemic events, a heightened risk of in-stent restenosis, and elevated cardiovascular mortality rates [7]. The management of these patients is further complicated by the necessity of administering antithrombotic therapy following ACS or PCI. Unfortunately, clinical trials assessing the safety and efficacy of antithrombotic agents in ACS or post-PCI settings frequently exclude individuals with chronic kidney disease, especially those with ESRD who are undergoing hemodialysis [8]. As a result, there is limited evidence supporting the safe and effective use of advanced cardiovascular therapies in these patients, leading to fewer evidence-based treatments being prescribed after an ACS [9].

Dual antiplatelet therapy, comprising Aspirin and P2Y12 inhibitors, is crucial in managing ACS, as it reduces platelet aggregation-associated risks [6]. However, this therapy carries an increased risk of bleeding, necessitating a personalized approach to determine the appropriate duration of therapy. Patients with end-stage renal disease have a higher bleeding risk, irrespective of therapy use, and are identified as a risk factor for bleeding after PCI in the ESC guidelines [6]. The Academic Research Consortium for High Bleeding Risk has also identified ESRD and dialysis as significant factors contributing to bleeding [10]. Consequently, achieving an optimal antiplatelet regimen for patients with ESRD remains challenging.

This narrative review article aims to critically examine the current evidence, guidelines, and controversies surrounding the use of dual anti-platelet therapy (DAPT) in ACS patients with ESRD. We will explore the delicate balance between reducing thrombotic events and minimizing bleeding complications in this vulnerable population. Furthermore, we will analyze available data from observational studies, subgroup analyses, and limited randomized controlled trials to elucidate the efficacy and safety profiles of various DAPT regimens in ESRD patients with ACS. Emerging strategies for optimizing treatment in this high-risk group will be examined, alongside a discussion on the potential of laboratory-guided precision medicine approaches. By synthesizing the latest evidence and clinical insights, this review aims to guide clinicians in making informed decisions to enhance outcomes for this complex patient cohort.

## 2. High Thrombotic Risk in ESRD

Patients with end-stage renal disease (ESRD) undergoing hemodialysis face a significantly elevated risk of thrombosis and hypercoagulable states. This heightened susceptibility stems from multiple mechanisms, including increased platelet aggregation, elevated levels of coagulation factors such as Fibrinogen and factor VIII:C, reduced anticoagulant activity of proteins C and S, and impaired fibrinolytic function. Additionally, elevated plasma lipoprotein(a) concentrations, high homocysteine levels, and the presence of lupus anticoagulant further contribute to this risk [11,12].

The progression of CKD is characterized by a pro-inflammatory state that significantly contributes to cardiovascular complications, such as ACS. This inflammatory process is marked by elevated levels of specific inflammatory markers and cytokines, including high-sensitivity C-reactive protein (hsCRP), fibrinogen, interleukin-1 (IL-1), interleukin-6 (IL-6), and tumor necrosis factor-alpha (TNFα). These markers are associated with the decline in kidney function and indicate systemic inflammation [11,13]. Furthermore, the process involves vascular inflammation and increased oxidative stress due to the excessive production of reactive oxygen and nitrogen species. The accumulation of uremic toxins in CKD patients further exacerbates this inflammatory state. Collectively, these pro-inflammatory conditions accelerate atherosclerosis, increase the risk of thrombosis, impair vascular function, and promote vascular calcification. These inflammatory indices are gaining increasing importance in the context of acute coronary syndromes [11,13,14].

The dialysis process itself exacerbates the thrombotic risk by inducing platelet degranulation and activation. Studies have demonstrated increased levels of P-selectin and fibrinogen receptor PAC-1 in platelets of dialysis patients [12]. Furthermore, endothelial injury and inflammation in ESRD compromise vascular integrity and antithrombotic properties, accelerating atherosclerosis and increasing plaque instability [14,15]. The damaged endothelium loses its ability to produce natural anticoagulants and becomes more prone to attracting platelets and inflammatory cells. This endothelial dysfunction, coupled with the accelerated atherosclerosis observed in ESRD patients, creates an environment highly conducive to thrombus formation [16], particularly in areas of plaque rupture, plaque erosion, or newly implanted stents.

**Figure 1:**
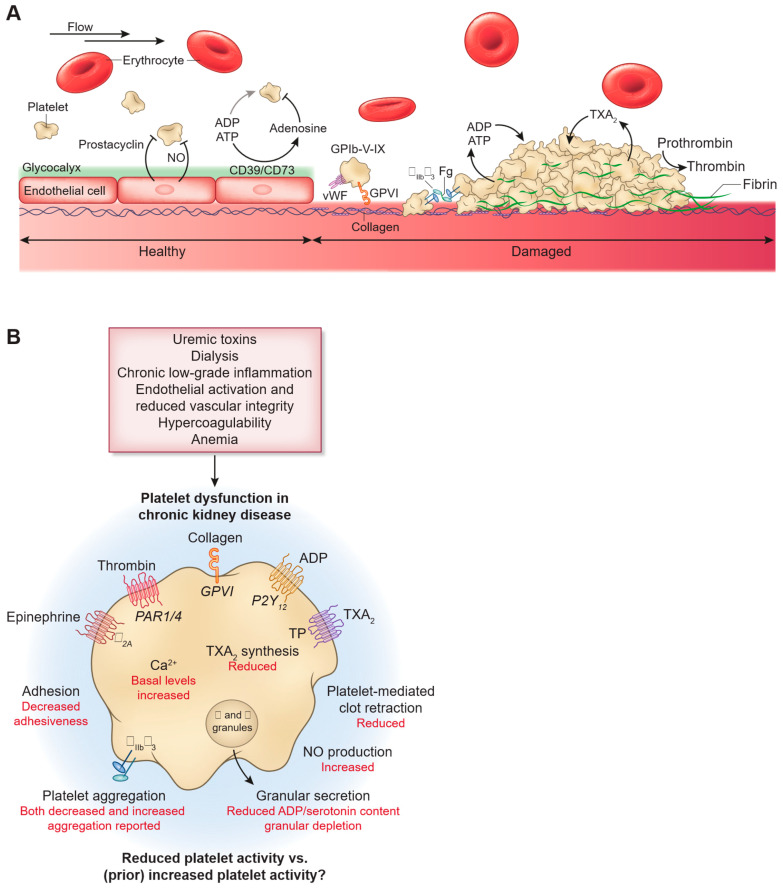
Impact of CKD and uremia on platelet dysfunction. Published with permission from CJASN [12]. (**A**) In healthy vasculature, the endothelium actively inhibits platelet activation through the release of nitric oxide (NO), prostacyclin, and ectonucleotidases (CD39/CD73), which degrade ATP and ADP, thereby limiting platelet recruitment. The endothelial glycocalyx and intact barrier prevent platelet adhesion. Following vascular injury, subendothelial collagen and von Willebrand factor (vWF) become exposed, triggering platelet adhesion via receptors such as GPIb-V-IX and GPVI. This activates a cascade involving granule secretion (e.g., ADP, serotonin), thromboxane A_2_ (TXA_2_) synthesis, and integrin activation, ultimately promoting aggregation and fibrin formation through thrombin, resulting in a stable thrombus. (**B**) In CKD, several pathological factors—uremic toxin accumulation, dialysis effects, persistent inflammation, vascular dysfunction, anemia, and a prothrombotic state—contribute to complex platelet abnormalities. These include reduced platelet adhesion, variable aggregation responses, decreased TXA_2_ production, impaired granule release with lower ADP and serotonin content, diminished clot retraction, elevated basal intracellular calcium, and increased NO synthesis. Together, these disturbances may underline the paradox of both bleeding and thrombotic risks observed in CKD.

Another critical factor in the hypercoagulable state of ESRD patients is the interaction of blood with external surfaces during hemodialysis. This interaction leads to alterations in extrinsic coagulation factors and tissue factor pathway inhibitors, resulting in the activation of the coagulation cascade [14]. These multifaceted factors collectively contribute to the high thrombotic risk observed in ESRD patients, necessitating careful management and monitoring (Figure 1).

## 3. Mechanisms of Higher Bleeding Risk in ESRD Patients

Despite increased thrombotic risk, individuals with end-stage renal disease (ESRD) exhibit higher bleeding tendencies, both spontaneously and under antiplatelet therapy. Patients with advanced kidney dysfunction have nearly doubled bleeding risk. Clinically, increased susceptibility to bleeding in these patients may present as symptoms such as gastrointestinal bleeding, subdural hematoma, epistaxis, retinal hemorrhage, hematuria, ecchymosis, purpura, bleeding from the gums, gingival bleeding, genital bleeding, hemoptysis, telangiectasia, hemarthrosis, and petechiae [12,17].

Platelet dysfunction in patients with severe renal impairment is a recognized issue. The disturbance of platelet α-granules, which exhibit an increased ATP/ADP ratio and reduced serotonin content, is a significant abnormality contributing to bleeding problems in these individuals [18,19]. Additionally, the release of ATP triggered by thrombin, along with elevated calcium levels and disrupted intracellular calcium flux in response to various stimuli, has been linked to platelet dysfunction and bleeding. Furthermore, the deregulation of arachidonic acid and disturbed prostaglandin metabolism in platelets of uremic patients impair the synthesis and/or release of thromboxane A2, which reduces platelet adhesion and aggregation, leading to a higher risk of bleeding. Moreover, fibrinogen fragments interfere with hemostasis by competitively binding to the glycoprotein (GP) IIb/IIIa receptors on platelets, decreasing platelet adhesion and aggregation potential. Additionally, the plasma of uremic patients contains higher levels of vasoactive substances, such as Nitric Oxide, which can affect platelet aggregation function [18,19,20].

Additionally, anemia of chronic renal disease plays a crucial role in increasing both bleeding and thrombotic risk. The erythrocyte lifespan and number decline as CKD progresses secondary to reduced erythropoietin in diseased kidneys [21]. A cohort study involving 74 non-smoking individuals with CKD investigated the lifespan of red blood cells (RBCs) at various stages of the condition [22]. The findings indicated a gradual decrease in RBC lifespan as CKD progressed. Specifically, the average RBC lifespans for CKD stages 1, 2, 3, 4, and 5 were 122 ± 50, 112 ± 26, 90 ± 32, 88 ± 28, and 60 ± 24 days, respectively. A notable reduction in RBC lifespan was evident from stage 3 onwards, with the lifespan at stage 5 being roughly half of that at stage 1. This decline was directly associated with decreasing hemoglobin levels (r = 0.372, *p* = 0.002). This reduction in RBCs reduces the displacement of platelets off the axial flow towards the vessel wall, impairing its function in hemostasis [22,23].

Dialysis has been shown to improve platelet function and reduce the risk of bleeding, although it does not eliminate it entirely. The interaction between blood and artificial surfaces during dialysis may cause chronic platelet activation, leading to platelet exhaustion and dysfunction. Additionally, research has found that plasma levels of NO inducers, such as tumor necrosis factor-a and interleukin-lb, increase during the dialysis process [20,24].

## 4. Balancing the Thrombotic and Bleeding Risks, Navigating the Challenges

The primary antiplatelet therapies used in patients with ACS are acetylsalicylic acid and P2Y12 inhibitors [6]. However, there is a lack of evidence regarding the most effective antiplatelet strategy for individuals with ESRD due to their limited representation or exclusion from major trials assessing such therapies [8]. As a result, the current knowledge about antiplatelet therapy in ESRD patients is primarily derived from underpowered post hoc subgroup analyses or large registries [12]. Moreover, the distinctive biological characteristics of these patients, which make them susceptible to both thrombosis and bleeding, further complicate the selection of the optimal antiplatelet therapy strategy [17,18]. According to the current ESC ACS guidelines, patients with ACS are recommended to receive aspirin in addition to potent P2Y12 inhibitors like ticagrelor and prasugrel [6]. In the absence of specific data, ESRD patients are typically treated with the same antiplatelet therapy as those with normal renal function [25].

### 4.1. Acetylsalicylic Acid

Aspirin functions by irreversibly inhibiting cyclooxygenase, thereby inhibiting thromboxane production. Its elimination mainly occurs through hepatic metabolism, but it is also excreted unchanged in urine, the extent of which depends on the dosage and urinary pH [26]. The efficacy of aspirin for patients with chronic kidney disease (CKD), including end-stage renal disease (ESRD), presenting with ACS, is well established (Table 1). A retrospective analysis of a prospective coronary care unit registry involving 1724 patients with ST-segment elevation myocardial infarction by McCullough et al. [27] has demonstrated that aspirin reduced in-hospital mortality by 64.3% to 80% across all quartiles of creatinine clearance (CrCl), including ESRD and dialysis patients. However, it was observed that patients with ESRD were less likely to receive aspirin compared to those without ESRD (67.0% vs. 82.4%, *p* < 0.001). Additionally, patients who did not receive aspirin upon admission had a higher likelihood of developing heart failure or cardiogenic shock [27,28,29]. Regarding the safety of low-dose aspirin in secondary prevention for coronary artery disease, two studies indicated no increased risk of major bleeding. In the First United Kingdom Heart and Renal Protection (UK-HARP) trial, an RCT involving 448 CKD patients, chronic hemodialysis or peritoneal dialysis patients, and previous kidney transplant recipients, no increased risk of major bleeding (defined as fatal or requiring hospitalization) was observed for CKD patients taking 100 mg aspirin (relative risk, 0.66; 95% CI, 0.19–2.31). However, there was a three-fold increase in the risk of minor bleeding (defined as epistaxis, ecchymosis, or bruising) (RR, 2.8; 95% CI, 1.5–5.3) [30]. Moreover, the Dialysis Outcomes and Practice Patterns Study (DOPPS), an observational study involving 28,320 patients, demonstrated no increased risk of gastrointestinal bleeding (RR, 1.01; 95% CI, 0.88–1.17) in individuals taking 100 mg/d of aspirin compared to those not taking aspirin [29,31].

### 4.2. Potent P2Y12 Inhibitors vs. Clopidogrel

Clopidogrel and Prasugrel are both P2Y12 receptor inhibitors, serving as prodrugs that selectively and irreversibly inhibit the P2Y12 receptor [35,36]. The blockade of the P2Y12 receptor occurs early in the platelet aggregation cascade, a crucial signaling pathway for platelet activation. Unlike Clopidogrel and Prasugrel, Ticagrelor is not a prodrug and does not necessitate metabolic conversion to an active form; it acts directly on P2Y12 receptors, and its effects are reversible [37]. Clopidogrel, on the other hand, becomes active through multiple activation steps by the cytochrome P450 system. As a result, Ticagrelor and Prasugrel exhibit more pronounced antiplatelet effects compared to Clopidogrel [38]. This superiority over Clopidogrel has been demonstrated in patients with ACS who also have chronic kidney disease (CKD) [15].

Clopidogrel remains the most frequently utilized P2Y12 inhibitor in patients with advanced renal disease who are undergoing dialysis [15,39]. However, it has several drawbacks, including a delayed onset of action, modest and variable platelet inhibition, and a high level of on-treatment platelet reactivity (HPR) observed in a significant proportion of patients [40]. Previous research has shown that patients with end-stage renal disease (ESRD) and those on hemodialysis (HD) therapy display higher platelet reactivity when treated with clopidogrel compared to individuals with normal kidney function, significantly reducing its effectiveness in these patients [41,42]. Moreover, a notable percentage of HD patients demonstrated non-responsiveness (resistance) to clopidogrel in another study [43]. Accumulating evidence has established that high on-treatment platelet reactivity (HPR) is linked to an increased risk of cardiovascular death and recurrent ischemic events, including myocardial infarction and stent thrombosis [42,43,44,45].

Several dedicated studies have investigated the pharmacodynamics and pharmacokinetics of potent oral P2Y12-ADP receptor antagonists in patients with chronic kidney disease (CKD) and end-stage renal disease (ESRD) (Table 1). Many of these studies involved comparisons with clopidogrel. Jeong et al. [43] conducted a study using a single-center, prospective, randomized, crossover design to examine the effects of ticagrelor compared to clopidogrel on platelet inhibition in patients undergoing hemodialysis. It was demonstrated that ticagrelor exhibited a more rapid and substantial platelet inhibition compared to clopidogrel, effectively overcoming high on-treatment platelet reactivity (HPR) in patients resistant to clopidogrel and undergoing maintenance hemodialysis [43]. Moreover, the rates of offset of the antiplatelet effect, as assessed by the inhibition of platelet aggregation IPA (5 and 20 mmol/L of ADP stimuli), were higher for ticagrelor than for clopidogrel in the 1 to 48 h after the last dose. A prospective two-center study by Alexopoulos et al. [46] involving 24 patients on hemodialysis showed that the maintenance dose of ticagrelor effectively reduced platelet reactivity in hemodialysis patients who had a poor response to clopidogrel. These patients had received regular hemodialysis for over six months and had ongoing clopidogrel treatment (75 mg/d). Platelet reactivity assessment was performed, and patients with ≥235 PRU (platelet reactivity unit) were considered to have high on-treatment platelet reactivity. Subsequently, these patients were administered ticagrelor alone for 15 days, and platelet reactivity was measured again. The baseline platelet reactivity for these patients was 310.4 ± 52.9 PRU and decreased significantly to 137.7 ± 77.9 PRU after ticagrelor treatment (*p* < 0.001) [46].

The potent P2Y12 inhibitors mentioned earlier demonstrated biological superiority over clopidogrel, leading to positive clinical outcomes in various trials. For instance, in the PLATelet inhibition and patient Outcomes (PLATO) trial, a multicenter randomized double-blind study, ticagrelor, when compared to clopidogrel, resulted in a decrease in the combined occurrence of cardiovascular death, nonfatal myocardial infarction, or stroke. However, it was also associated with an increased incidence of bleeding not related to procedures [32]. In a subset analysis of the PLATO trial, the benefits of ticagrelor were even more pronounced in patients with all degrees of chronic kidney disease (CKD), including advanced stages, showing a relative reduction of 23% in the primary ischemic end point [32].

Furthermore, in the United States Renal Data System registry of Medicare beneficiaries with ESRD registry data highlighted the effectiveness and safety of clopidogrel, prasugrel, and ticagrelor. The study included individuals who received new prescriptions for P2Y12 inhibitors and were tracked until death or censoring. The primary focus was on P2Y12 inhibitor assignment as the exposure variable and death as the primary outcome. Secondary outcomes encompassed cardiovascular (CV) death, coronary revascularization, and gastrointestinal (GI) hemorrhage. The results demonstrated that prasugrel exhibited superior efficacy compared to both clopidogrel and ticagrelor, as evidenced by a statistically significant reduction in mortality risk (adjusted hazard ratio [HR] = 0.82; 95% confidence interval [CI]: 0.73–0.93 and 0.78; 95% CI: 0.64–0.95, respectively). Furthermore, prasugrel was associated with a decreased risk of coronary revascularization relative to clopidogrel (HR = 0.91; 95% CI: 0.86–0.96) [47].

The study conducted by Edfors et al., based on the SWEDEHEART Registry data [33], aimed to compare the efficacy of ticagrelor and clopidogrel in chronic kidney disease (CKD) patients undergoing PCI for ACS. The total patient cohort consisted of 45,206 individuals, with 1735 of them having an estimated glomerular filtration rate (eGFR) less than 30. The primary outcome measured was a composite of death, myocardial infarction (MI), or stroke, while the secondary outcome focused on rehospitalization due to bleeding. The unadjusted 1-year event rate for the composite endpoint of death, MI, or stroke in patients with an eGFR less than 30 was 48.0% for those treated with ticagrelor and 64.0% for those treated with clopidogrel. After adjustment, ticagrelor was found to be associated with a lower 1-year risk of the composite outcome when compared with clopidogrel for all patients. For those with an eGFR less than 30, the hazard ratio was 0.95 (95% confidence interval: 0.69 to 1.29), and the *p*-value for interaction was 0.55. However, it is important to note that patients treated with ticagrelor had a higher risk of bleeding compared to those treated with clopidogrel, and the hazard ratio for the eGFR-less-than-30 group was 1.79 (95% confidence interval: 1.00 to 3.21)

Interestingly, in medically managed ACS patients with CKD, including those with severe CKD, prasugrel did not demonstrate any advantages when compared to clopidogrel combined with aspirin. The TRILOGY ACS trial (Targeted Platelet Inhibition to Clarify the Optimal Strategy to Medically managed ACS) involved a randomized comparison of prasugrel and clopidogrel therapy in conjunction with aspirin among medically managed ACS patients. The main study assessed the primary endpoint, which was a combination of cardiovascular death, myocardial infarction, and stroke, and found no significant difference between prasugrel and clopidogrel (13.9% versus 16.0%; *p* = 0.20). Moreover, the rates of severe and intracranial bleeding were comparable in both treatment groups (TIMI major 2.1% versus 1.5%; *p* = 0.27). A subgroup analysis was conducted, revealing that patients with moderate or severe CKD faced an elevated risk of both ischemic and bleeding events. However, when comparing outcomes between prasugrel and clopidogrel in these subgroups, no discernible differences were observed [26]

A recent meta-analysis [34] investigated the clinical effectiveness and safety of various antiplatelet therapy regimens that exhibit potent platelet-inhibition activity compared to a standard dose of clopidogrel-based dual antiplatelet therapy (DAPT) in individuals with CKD, including those with ESRD undergoing dialysis. The study compared the outcomes of doubled loading dose (LD) clopidogrel-based DAPT, doubled maintenance dose (MD) clopidogrel-based DAPT, prasugrel-based DAPT, and ticagrelor-based DAPT. The findings revealed that antiplatelet therapy regimens with enhanced platelet inhibition beyond the standard clopidogrel-based DAPT significantly improved clinical outcomes in patients with ACS and CKD, including ESRD and dialysis patients. These improvements included reduced all-cause mortality (relative risk [RR] 0.67, *p* = 0.003), major adverse cardiovascular events (MACE) (RR 0.79, *p* < 0.00001), and myocardial infarction (MI) (RR 0.28, *p* = 0.0007) without an increase in major bleeding (RR 1.14, *p* = 0.33). However, a subgroup analysis demonstrated that the intervention led to a substantial increase in both major and minor bleeding in patients with severe CKD (eGFR < 30 mL/min) or those on hemodialysis (RR 1.30; 95% CI 1.09, 1.55; *p* = 0.002).

### 4.3. The Duration of DAPT After PCI

Determining the optimal duration of DAPT following coronary revascularization through PCI poses a challenge for clinicians dealing with patients with ESRD. According to current ESC guidelines, the recommended DAPT duration for ACS patients who received drug-eluting stents (DES) is typically 12 months [6]. However, ESRD patients are often excluded from large randomized clinical trials, making it challenging to establish specific recommendations for this group [8]. Park et al. [48] conducted a population-based trial to investigate the ischemic and bleeding outcomes of prolonged DAPT in over 5000 dialysis patients who underwent DES implantation, with most of them having the DES placed after an ACS episode. The study compared continued DAPT with discontinued DAPT using landmark analyses, evaluating outcomes at 12, 15, and 18 months after DES implantation. The primary outcome measured was major adverse cardiovascular events (MACEs), defined as a composite of mortality, nonfatal myocardial infarction, coronary revascularization, and stroke. A longer DAPT duration was associated with a significant reduction in MACEs, Specifically, continued DAPT reduced the hazard of MACE at 12 months (HR = 0.74, 95% CI 0.61–0.90; *p* = 0.003), 15 months (HR = 0.78, 95% CI 0.64–0.96; *p* = 0.019), and 18 months (HR = 0.79, 95% CI 0.63–0.99; *p* = 0.041) after DES implantation. Notably, this reduction in MACE was achieved without a significant increase in major bleeding at any of these time points. The incidence of major bleeding was consistently lower than that of MACE across all time points. These findings suggest that prolonged DAPT may be beneficial for dialysis patients who have undergone DES implantation, as it reduces the risk of adverse cardiovascular events without substantially increasing the risk of major bleeding.

### 4.4. Alternative Anti-Thrombotic Regimens

Emerging strategies for antithrombotic therapy, specifically monotherapy following PCI in patients with ACS, aim to address the balance between thrombotic and bleeding risks. Ticagrelor monotherapy has shown potential to reduce bleeding risk without compromising ischemic protection, which may be particularly beneficial for patients with end-stage renal disease. A recent meta-analysis by Alagna et al. [49], encompassing five randomized controlled trials with 32,393 patients, indicates that ticagrelor monotherapy following short-duration dual antiplatelet therapy (DAPT) offers significant advantages for patients undergoing PCI after ACS. This approach significantly reduced major adverse cardiovascular and cerebrovascular events (MACCE) by 12% (RR: 0.88; 95% CI: 0.77 to 0.99; *p* = 0.04) and major bleeding by 47% (RR: 0.53; 95% CI: 0.37 to 0.77; *p* = 0.0008) compared to extended DAPT. Notably, ticagrelor monotherapy also decreased all-cause mortality by 18% (RR: 0.82; 95% CI: 0.67 to 0.99; *p* = 0.04) and cardiovascular mortality by 32% (RR: 0.68; 95% CI: 0.49 to 0.94; *p* = 0.02). The incidence of myocardial infarction, stent thrombosis, and stroke remained comparable between the two strategies. Overall, net adverse clinical events (NACE) were 27% lower with ticagrelor monotherapy (RR: 0.73; 95% CI: 0.63 to 0.85; *p* <0.0001). These findings suggest that de-escalation to ticagrelor monotherapy after a short course of DAPT effectively mitigates bleeding risk without compromising ischemic protection in ACS patients undergoing PCI, which can be particularly advantageous for patients with end-stage renal disease, offering a more balanced approach to antiplatelet therapy.

Cangrelor, an intravenous P2Y12 inhibitor, has shown promising results in patients with ACS and CKD. In a real-world registry study [50], Cangrelor was predominantly administered to ACS patients with complex clinical presentations. This observational study assessed the use of cangrelor in 686 patients with ACS undergoing PCI. The cangrelor cohort (*n* = 198) exhibited a higher-risk clinical profile, characterized by a greater prevalence of left ventricular ejection fraction <30% and cardiogenic shock. Additionally, the cangrelor group had a higher prevalence of CKD compared to the non-cangrelor group. Initially, the cangrelor group experienced higher in-hospital mortality (12.1% vs. 4.9%, *p* = 0.001). However, following propensity score matching (*n* = 356), mortality rates were comparable between the groups. Notably, in the matched population, cangrelor use was associated with a significant reduction in in-hospital definite stent thrombosis (0% vs. 2.8%, *p* = 0.030) without an increase in bleeding risk. The study suggests potential benefits of cangrelor in high-risk ACS patients, particularly in reducing stent thrombosis. Nonetheless, the authors caution that due to the study’s observational nature and limited sample size, these findings should be considered hypothesis-generating and warrant confirmation through larger randomized trials involving high-risk patients, especially those with chronic kidney disease.

Low-dose rivaroxaban could potentially offer a future strategy for reducing ischemic risk without significantly increasing bleeding risk. The ATLAS ACS 2-TIMI 51 study [51] assessed low-dose rivaroxaban (2.5 mg or 5 mg twice daily) alongside standard antiplatelet therapy with Aspirin and Clopidogrel in patients who had recently experienced ACS, excluding those with severe renal impairment. The study found that rivaroxaban significantly reduced the primary efficacy endpoint compared to placebo, with the 2.5 mg twice-daily dosage showing a survival benefit. Although these cardiovascular benefits were associated with an increased risk of major bleeding and intracranial hemorrhage, there was no significant rise in fatal bleeding. This indicates that adding very-low-dose rivaroxaban to standard antiplatelet therapy may enhance cardiovascular outcomes with a manageable bleeding risk. Further research is necessary to evaluate low-dose rivaroxaban in patients with ESRD, potentially providing a means to reduce ischemic risk without substantially increasing bleeding risk.

## 5. Laboratory Guided Precision Medicine Approaches

Platelet function tests (PFT), such as VerifyNow and platelet reactivity unit (PRU) measurements, have demonstrated potential utility in evaluating the response to antiplatelet therapy, particularly in high-risk populations, including those with ESRD [52]. In patients with CKD, including those with ESRD, PFT have indicated increased baseline platelet activation and a diminished response to dual antiplatelet therapy compared to patients without renal insufficiency [53]. Individualized antiplatelet therapy approaches, based on PFT and genetic testing, have been investigated in several randomized trials to optimize treatment for patients with acute coronary syndrome [54]. These strategies aim to balance the reduction in ischemic events with the risk of bleeding complications by de-escalating antiplatelet therapy based on PFT and genetic testing [55].

The TROPICAL-ACS trial involved the randomization of 2610 patients to receive either standard dual antiplatelet therapy (DAPT) with prasugrel or a de-escalation strategy guided by platelet function testing (PFT). The primary endpoint was a composite measure of cardiovascular death, myocardial infarction, stroke, and bleeding at one year. The study concluded that PFT-guided de-escalation was non-inferior to standard DAPT with respect to the primary endpoint (7% vs. 9%, *p* = 0.0004 for non-inferiority; hazard ratio: 0.81, 95% CI: 0.62–1.06, *p* = 0.12 for superiority). Although the trial indicated a 15% reduction in the risk of major bleeding with the guided de-escalation strategy, this reduction did not achieve statistical significance [56].

The TAILOR-PCI trial was a large-scale randomized clinical study designed to assess the effectiveness of a genotype-guided approach to antiplatelet therapy in patients with acute coronary syndrome (ACS) undergoing PCI. A total of 5302 participants were randomly assigned to receive either the standard dual antiplatelet therapy (DAPT) consisting of aspirin and clopidogrel or a personalized treatment strategy based on CYP2C19 genetic testing to guide the selection of a P2Y12 inhibitor. The primary outcome measured was a composite of cardiovascular death, myocardial infarction, stroke, stent thrombosis, and severe recurrent ischemia within 12 months. The results indicated that the genotype-guided strategy was not inferior to standard DAPT regarding the primary outcome (4.0% vs. 5.9%, HR: 0.66, 95% CI: 0.43–1.02, *p* = 0.06). Although the data suggested a potential advantage for the genotype-guided method, the difference did not achieve statistical significance. Furthermore, the incidence of major and minor bleeding events was similar across both treatment groups [57].

While these personalized approaches offer insights into patients’ responses to antiplatelet medications, their clinical benefits may not yet fully achieve the anticipated advantages over uniform de-escalation strategies. The unexpected results indicate that laboratory-guided precision medicine approaches in this context may require further refinement. Additional research is necessary to optimize these individualized strategies and determine their most effective implementation in clinical practice, particularly for high-risk patients such as those with ESRD [54].

## 6. Conclusions

While advancements have been made in the management of ACS, there are unique challenges when it comes to treating ACS patients with ESRD. The use of potent P2Y12 inhibitors has shown promise in reducing ischemic risk but comes with an increased bleeding risk. Alternative regimens like ticagrelor monotherapy after short-term DAPT may offer a more balanced approach. The optimal duration of DAPT remains controversial, highlighting the need for individualized strategies. Laboratory-guided precision medicine approaches show potential but require further refinement. Future research should focus on optimizing individualized treatment strategies, exploring novel therapies, and increasing the inclusion of ESRD patients in clinical trials to establish evidence-based guidelines for this high-risk population.

## Figures and Tables

**Table 1:** Trials comparing antiplatelet therapy outcomes in CKD populations.

Trial	Study Population	MACE Outcome (CV Death/MI/Stroke)	Bleeding
McCullough 2002 [27]	1724 STEMI patients (registry); Aspirin + β-blocker vs. none, stratified by CrCl	Marked benefit in terms of mortality. In-hospital MACE (driven by death) was much lower with ASA + BB across all CKD strata. Mortality RR reduction was ~64–80% in CKD patients on ASA + BB (vs. no therapy)	Bleeding not reported (acute registry; no significant excess noted in-hospital).
UK-HARP-I 2005 [30]	448 CKD patients (predialysis, dialysis, transplant—RCT of aspirin 100 mg vs. placebo (1 yr))	Not powered for MACE (no significant difference observed)	Major bleeding: no increase with aspirin (2% vs. 3%, NS). Minor bleeding: 3-fold higher with aspirin (15% vs. 5%, *p* < 0.001)
DOPPS 2007 [31]	28,320 hemodialysis patients (observational; Aspirin vs. no Aspirin)	No net CV benefit. Aspirin did not lower composite cardiac events	No increase in major GI bleeding noted with aspirin (no significant hemorrhagic risk observed)
PLATO 2010 [32]	CKD subgroup = CrCl <60 mL/min (*n* = 3237)	Significant MACE reduction. Ticagrelor vs. clopidogrel lowered 12 month CV death/MI/stroke in CKD (17.3% vs. 22.0%; HR 0.77, 95% CI 0.65–0.90), an absolute risk reduction of ~4.7%.	Major bleeding: no significant difference (15.1% vs. 14.3%, HR 1.07, *p* = NS) in CKD. No increase in fatal bleeds; slight, non-significant ↑ in non-CABG major bleeds
TRILOGY-ACS 2012 [26]	Patients with NSTE-ACS managed medically without revascularization; CKD subgroup included	No significant difference in MACE between prasugrel and clopidogrel in CKD patients (13.9% versus 16.0%; *p* = 0.20)	Bleeding rates similar between prasugrel and clopidogrel in CKD subgroup (TIMI major 2.1% versus 1.5%; *p* = 0.27)
Edfors et al., 2018 [33]	45,206 post-MI patients on DAPT (ticagrelor vs. clopidogrel), stratified by eGFR	Lower MACE with ticagrelor in moderate CKD. One-year death/MI/stroke rate was lower for ticagrelor vs. clopidogrel in eGFR 30–60 (adj. HR 0.82, 95% CI 0.70–0.97). In severe CKD (eGFR < 30), no significant benefit (HR 0.95, 95% CI 0.69–1.29)	Major bleeding (requiring hospitalization): no significant difference in moderate CKD (HR 1.13, 95% CI 0.84–1.51) but ↑ trend in severe CKD on ticagrelor (HR 1.79, 95% CI 1.00–3.21)
Meta-analysis (2020)—P2Y12 Inhibitors in CKD [34]	Pooled ACS trial data in CKD patients (prasugrel or ticagrelor vs. clopidogrel)	Improved outcomes. Potent P2Y12 inhibitors associated with lower MACE (especially reduced MI and mortality) in CKD	Bleeding: No significant increase in major bleeding with prasugrel or ticagrelor in CKD (vs. clopidogrel)

Abbreviations: CKD—chronic kidney disease; NSTE-ACS—non–ST-elevation acute coronary syndrome; MACE—major adverse cardiovascular events; CV—cardiovascular; MI—myocardial infarction; ASA—aspirin; RR—relative risk; HR—hazard ratio; CI—confidence interval; NS—not significant; RCT—randomized controlled trial.

## Abbreviations

ACS: Acute Coronary Syndrome; ESRD: End-Stage Renal Disease; ESC: European Society of Cardiology; CKD: Chronic Kidney Disease; CAD: Coronary Artery Disease; eGFR: estimated Glomerular Filtration Rate; PCI: Percutaneous Coronary Intervention; DAPT: Dual Antiplatelet Therapy; MACCE: Major Adverse Cardiovascular and Cerebrovascular Events; MACE: Major Adverse Cardiovascular Events; MI: Myocardial Infarction; CV: Cardiovascular; NSTE-ACS: Non-ST Elevation Acute Coronary Syndrome; ASA: Aspirin; RR: Relative Risk; HR: Hazard Ratio; CI: Confidence Interval; NS: Not Significant; RCT: Randomized Controlled Trial; HD: Hemodialysis; ESKD: End-Stage Kidney Disease; PRU: Platelet Reactivity Unit; HPR: High on-treatment Platelet Reactivity; LD: Loading Dose; MD: Maintenance Dose; NACE: Net Adverse Clinical Events; PFT: Platelet Function Test.

## References

[1] Matsushita K., Ballew S.H., Wang A.Y., Kalyesubula R., Schaeffner E., Agarwal R. (2022). Epidemiology and risk of cardiovascular disease in populations with chronic kidney disease. Nat. Rev. Nephrol..

[2] Go A.S., Bansal N., Chandra M., Lathon P.V., Fortmann S.P., Iribarren C., Hsu C.-Y., Hlatky M.A. (2011). Chronic kidney disease and risk for presenting with acute myocardial infarction versus stable exertional angina in adults with coronary heart disease. J. Am. Coll. Cardiol..

[3] Wang Y., Gao L. (2022). Inflammation and Cardiovascular Disease Associated with Hemodialysis for End-Stage Renal Disease. Front Pharmacol..

[4] Echefu G., Stowe I., Burka S., Basu-Ray I., Kumbala D. (2023). Pathophysiological concepts and screening of cardiovascular disease in dialysis patients. Front Nephrol..

[5] Charytan D., Kuntz R.E., Mauri L., DeFilippi C. (2007). Distribution of coronary artery disease and relation to mortality in asymptomatic hemodialysis patients. Am. J. Kidney Dis..

[6] Byrne R., Asteggiano R., Marjeh M.Y.B., Rocca B., Zeppenfeld K., Geisler T., Dan G.-A., Ryödi E., Coughlan J.J., Wiseth R. (2023). 2023 ESC Guidelines for the management of acute coronary syndromes. Eur. Heart J..

[7] Jakubiak G.K., Cieślar G., Stanek A., Pawlas N. (2021). Pathogenesis and Clinical Significance of In-Stent Restenosis in Patients with Diabetes. Int. J. Environ. Res. Public. Health.

[8] Charytan D., Kuntz R.E. (2006). The exclusion of patients with chronic kidney disease from clinical trials in coronary artery disease. Kidney Int..

[9] Fox C.S., Muntner P., Chen A.Y., Alexander K.P., Roe M.T., Cannon C.P., Saucedo J.F., Kontos M.C., Wiviott S.D. (2010). Use of evidence-based therapies in short-term outcomes of ST-segment elevation myocardial infarction and non-ST-segment elevation myocardial infarction in patients with chronic kidney disease: A report from the National Cardiovascular Data Acute Coronary Treatment and Intervention Outcomes Network registry. Circulation.

[10] Urban P., Mehran R., Colleran R., Angiolillo D.J., Byrne R.A., Capodanno D., Cuisset T., Cutlip D., Eerdmans P., Eikelboom J. (2019). Defining High Bleeding Risk in Patients Undergoing Percutaneous Coronary Intervention. Circulation.

[11] Chermiti R., Burtey S., Dou L. (2024). Role of Uremic Toxins in Vascular Inflammation Associated with Chronic Kidney Disease. J. Clin. Med..

[12] Baaten C.C.F.M.J., Schröer J.R., Floege J., Marx N., Jankowski J., Berger M., Noels H. (2022). Platelet Abnormalities in CKD and Their Implications for Antiplatelet Therapy. Clin. J. Am. Soc. Nephrol..

[13] Li X., Lindholm B. (2022). Cardiovascular Risk Prediction in Chronic Kidney Disease. Am. J. Nephrol..

[14] Trimarchi G., Pizzino F. (2024). An in-depth look at electrolytes in acute heart failure: The role of sodium-to-chloride ratio. Int. J. Cardiol..

[15] Bonello L., Angiolillo D.J., Aradi D., Sibbing D. (2018). P2Y_12_-ADP Receptor Blockade in Chronic Kidney Disease Patients With Acute Coronary Syndromes. Circulation.

[16] Kadowaki T., Maegawa H., Yabe D., Wada J., Node K., Murohara T., Watada H. (2022). Interconnection between cardiovascular, renal and metabolic disorders: A narrative review with a focus on Japan. Diabetes Obes. Metab..

[17] Burlacu A., Genovesi S., Ortiz A., Kanbay M., Rossignol P., Banach M., Małyszko J., Goldsmith D., Covic A. (2017). The quest for equilibrium: Exploring the thin red line between bleeding and ischaemic risks in the management of acute coronary syndromes in chronic kidney disease patients. Nephrol. Dial. Transplant..

[18] Qiu Z., Pang X., Xiang Q., Cui Y. (2023). The Crosstalk between Nephropathy and Coagulation Disorder: Pathogenesis, Treatment, and Dilemmas. J. Am. Soc. Nephrol..

[19] Galbusera M., Remuzzi G., Boccardo P. (2009). Treatment of bleeding in dialysis patients. Semin. Dial..

[20] Boccardo P., Remuzzi G., Galbusera M. (2004). Platelet dysfunction in renal failure. Semin. Thromb. Hemost..

[21] Portolés J., Broseta J.J., Cases A., Martín L. (2021). Anemia in Chronic Kidney Disease: From Pathophysiology and Current Treatments, to Future Agents. Front. Med..

[22] Li J.-H., Luo J.-F., Jiang Y., Ma Y.-J., Ji Y.-Q., Zhu G.-L., Zhou C., Chu H.-W., Zhang H.-D. (2019). Red Blood Cell Lifespan Shortening in Patients with Early-Stage Chronic Kidney Disease. Kidney Blood Press. Res..

[23] Weisel J.W., Litvinov R.I. (2019). Red blood cells: The forgotten player in hemostasis and thrombosis. J. Thromb. Haemost..

[24] Gäckler A., Rohn H., Lisman T., Benkö T., Witzke O., Kribben A., Saner F.H. (2019). Evaluation of hemostasis in patients with end-stage renal disease. PLoS ONE.

[25] Szummer K., Lundman P., Jacobson S.H., Schön S., Lindbäck J., Stenestrand U., Wallentin L., Jernberg T., Swedeheart (2010). Relation between renal function, presentation, use of therapies and in-hospital complications in acute coronary syndrome: Data from the SWEDEHEART register. J. Intern. Med..

[26] Melloni C., Cornel J.H., Hafley G., Neely M.L., Clemmensen P., Zamoryakhin D., Prabhakaran D., White H.D., Fox K.A., Ohman E.M. (2016). Impact of chronic kidney disease on long-term ischemic and bleeding outcomes in medically managed patients with acute coronary syndromes: Insights from the TRILOGY ACS Trial. Eur. Heart J. Acute Cardiovasc. Care.

[27] McCullough P.A., Sandberg K.R., Borzak S., Hudson M.P., Garg M., Manley H.J. (2002). Benefits of aspirin and beta-blockade after myocardial infarction in patients with chronic kidney disease. Am. Heart J..

[28] Jain N., Hedayati S.S., Sarode R., Banerjee S., Reilly R.F. (2013). Antiplatelet therapy in the management of cardiovascular disease in patients with CKD: What is the evidence?. Clin. J. Am. Soc. Nephrol..

[29] Berger A.K., Duval S., Krumholz H.M. (2003). Aspirin, beta-blocker, and angiotensin-converting enzyme inhibitor therapy in patients with end-stage renal disease and an acute myocardial infarction. J. Am. Coll. Cardiol..

[30] Baigent C., Landray M., Leaper C., Altmann P., Armitage J., Baxter A., Cairns H.S., Collins R., Foley R.N., Frighi V. (2005). First United Kingdom Heart and Renal Protection (UK-HARP-I) study: Biochemical efficacy and safety of simvastatin and safety of low-dose aspirin in chronic kidney disease. Am. J. Kidney Dis..

[31] Patrono C., García Rodríguez L.A., Landolfi R., Baigent C. (2005). Low-dose aspirin for the prevention of atherothrombosis. N. Engl. J. Med..

[32] James S., Budaj A., Aylward P., Buck K.K., Cannon C.P., Cornel J.H., Harrington R.A., Horrow J., Katus H., Keltai M. (2010). Ticagrelor versus clopidogrel in acute coronary syndromes in relation to renal function: Results from the Platelet Inhibition and Patient Outcomes (PLATO) trial. Circulation.

[33] Edfors R., Sahlén A., Szummer K., Renlund H., Evans M., Carrero J.-J., Spaak J., James S.K., Lagerqvist B., Varenhorst C. (2018). Outcomes in patients treated with ticagrelor versus clopidogrel after acute myocardial infarction stratified by renal function. Heart.

[34] Park S., Choi Y.J., Kang J.E., Kim M.G., Geum M.J., Kim S.D., Rhie S.J. (2021). P2Y12 Antiplatelet Choice for Patients with Chronic Kidney Disease and Acute Coronary Syndrome: A Systematic Review and Meta-Analysis. J. Pers. Med..

[35] Mullangi R., Srinivas N.R. (2009). Clopidogrel: Review of bioanalytical methods, pharmacokinetics/pharmacodynamics, and update on recent trends in drug-drug interaction studies. Biomed. Chromatogr..

[36] Gimbel M., Qaderdan K., Willemsen L., Hermanides R., Bergmeijer T., de Vrey E., Heestermans T., Gin M.T.J., Waalewijn R., Hofma S. (2020). Clopidogrel versus ticagrelor or prasugrel in patients aged 70 years or older with non-ST-elevation acute coronary syndrome (POPular AGE): The randomised, open-label, non-inferiority trial. Lancet.

[37] Shah R.P., Shafiq A., Hamza M., Maniya M.T., Duhan S., Keisham B., Patel B., Alamzaib S.M., Yashi K., Uppal D. (2023). Ticagrelor Versus Prasugrel in Patients With Acute Coronary Syndrome: A Systematic Review and Meta-Analysis. Am J Cardiol..

[38] Kamran H., Jneid H., Kayani W.T., Virani S.S., Levine G.N., Nambi V., Khalid U. (2021). Oral Antiplatelet Therapy After Acute Coronary Syndrome: A Review. JAMA.

[39] De Filippo O., D’Ascenzo F., Raposeiras-Roubin S., Abu-Assi E., Peyracchia M., Bocchino P.P., Kinnaird T., Ariza-Solé A., Liebetrau C., Manzano-Fernández S. (2020). P2Y12 inhibitors in acute coronary syndrome patients with renal dysfunction: An analysis from the RENAMI and BleeMACS projects. Eur. Heart J. Cardiovasc. Pharmacother..

[40] Müller I., Besta F., Schulz C., Massberg S., Schönig A., Gawaz M. (2003). Prevalence of clopidogrel non-responders among patients with stable angina pectoris scheduled for elective coronary stent placement. Thromb. Haemost..

[41] Woo J.S., Kim W., Lee S.R., Jung K.H., Kim W.S., Lew J.H., Lee T.W., Lim C.K. (2011). Platelet reactivity in patients with chronic kidney disease receiving adjunctive cilostazol compared with a high-maintenance dose of clopidogrel: Results of the effect of platelet inhibition according to clopidogrel dose in patients with chronic kidney disease (PIANO-2 CKD) randomized study. Am. Heart J..

[42] Ye Z., Wang Q., Ullah I., Lin Q., Wu T., Yang M., Fan Y., Dong Z., Wang T., Teng J. (2024). Impact of hemodialysis on efficacies of the antiplatelet agents in coronary artery disease patients complicated with end-stage renal disease. J. Thromb. Thrombolysis.

[43] Jeong K.H., Cho J.H., Woo J.S., Kim J.B., Kim W.-S., Lee T.W., Kim K.S., Ihm C.G., Kim W. (2015). Platelet reactivity after receiving clopidogrel compared with ticagrelor in patients with kidney failure treated with hemodialysis: A randomized crossover study. Am. J. Kidney Dis..

[44] Morel O., El Ghannudi S., Jesel L., Radulescu B., Meyer N., Wiesel M.-L., Caillard S., Campia U., Moulin B., Gachet C. (2011). Cardiovascular mortality in chronic kidney disease patients undergoing percutaneous coronary intervention is mainly related to impaired P2Y12 inhibition by clopidogrel. J. Am. Coll. Cardiol..

[45] Rubin G.A., Kirtane A.J., Chen S., Redfors B., Weisz G., Baber U., Zhang Y., Stuckey T.D., Witzenbichler B., Rinaldi M.J. (2020). Impact of high on-treatment platelet reactivity on outcomes following PCI in patients on hemodialysis: An ADAPT-DES substudy. Catheter. Cardiovasc. Interv..

[46] Alexopoulos D., Xanthopoulou I., Plakomyti T.E., Goudas P., Koutroulia E., Goumenos D. (2012). Ticagrelor in clopidogrel-resistant patients undergoing maintenance hemodialysis. Am. J. Kidney Dis..

[47] Jain N., Phadnis M.A., Hunt S.L., Dai J., Shireman T.I., Davis C.L., Mehta J.L., Rasu R.S., Hedayati S.S. (2021). Comparative Effectiveness and Safety of Oral P2Y12 Inhibitors in Patients on Chronic Dialysis. Kidney Int. Rep..

[48] Park S., Kim Y., Jo H.A., Lee S., Kim M.-S., Yang B.R., Lee J., Han S.S., Lee H., Lee J.P. (2020). Clinical outcomes of prolonged dual antiplatelet therapy after coronary drug-eluting stent implantation in dialysis patients. Clin. Kidney J..

[49] Alagna G., Trimarchi G., Cascone A., Villari A., Cavolina G., Campanella F., Micari A., Taverna G., Andò G. (2025). Effectiveness and Safety of Ticagrelor Monotherapy After Short-Duration Dual Antiplatelet Therapy in PCI Patients: A Systematic Review and Meta-Analysis. Am. J. Cardiol..

[50] Pepe M., Carella M.C., Nestola P.L., Napoli G., Giordano S., Cirillo P., Giordano A., Carulli E., Bartolomucci F., Tritto R. (2023). Comparative effectiveness of Cangrelor in patients with acute coronary syndrome undergoing percutaneous coronary intervention: An observational investigation from the M.O.Ca. registry. Sci. Rep..

[51] Mega J.L., Verheugt F.W.A., Plotnikov A.N., Burton P., Braunwald E., Bode C., Bassand J.-P., Sun X., Bhatt D.L., Cook-Bruns N. (2012). Rivaroxaban in Patients with a Recent Acute Coronary Syndrome. New Engl. J. Med..

[52] Aradi D., Sibbing D., Gross L. (2016). Platelet Function Testing in Patients on Antiplatelet Medications. Semin. Thromb. Hemost..

[53] Gremmel T., Panzer S., Seidinger D., Kopp C.W., Steiner S., Muller M., Koppensteiner R. (2013). Chronic kidney disease is associated with increased platelet activation and poor response to antiplatelet therapy. Nephrol. Dial. Transplant..

[54] El Abdallaoui O.E.A., Szabó D., Lukács R., Komócsi A., Tornyos D. (2023). Individualized or Uniform De-Escalation Strategies for Antiplatelet Therapy in Acute Coronary Syndrome: A Review of Clinical Trials with Platelet Function Testing and Genetic Testing-Based Protocols. Int. J. Mol. Sci..

[55] Angiolillo D.J., Berg J.T., Gibson C.M., Waksman R., Cavallari L.H., Siller-Matula J.M., Capodanno D., Alexopoulos D., Franchi F., Bonello L. (2024). International Consensus Statement on Platelet Function and Genetic Testing in Percutaneous Coronary Intervention: 2024 Update. JACC Cardiovasc. Interv..

[56] Sibbing D., Zweiker R., Kääb S., Kovács A., Komócsi A., Aradi D., Müller K., Ungi I., Mügge A., Ili R. (2017). Guided de-escalation of antiplatelet treatment in patients with acute coronary syndrome undergoing percutaneous coronary intervention (TROPICAL-ACS): A randomised, open-label, multicentre trial. Lancet.

[57] Pereira N.L., Gordon P., Weinshilboum R., Jeong M.H., Hasan A., Lennon R., Farkouh M.E., Bailey K., Bae J., So D. (2020). Effect of Genotype-Guided Oral P2Y12 Inhibitor Selection vs. Conventional Clopidogrel Therapy on Ischemic Outcomes After Percutaneous Coronary Intervention. JAMA.

